# Longitudinal Changes in Functioning and Support After Initial Survey for Individuals With Severe Mental Illness

**DOI:** 10.1093/geroni/igaa057.311

**Published:** 2020-12-16

**Authors:** Deborah Finkel, Per Bulöw, Pia Bulöw, Cristina Joy Torgé, Monika Wilińska, Magnus Jegermalm, Marie Ernsth Bravell

**Affiliations:** 1 Indiana University Southeast, New Albany, Indiana, United States; 2 Jönköping University, Jönköping, Sweden

## Abstract

After the Swedish Mental Health Care reform was enacted in 1995, many individuals with severe mental illness were released from long-term care institutions. As a result, they had new living and support situations. Following 236 individuals over time supported investigation of the effectiveness of social support services in caring for these individuals. Surveys were conducted in 1996, 2001, 2006, and 2011 and annual data were available from national registries. Primary diagnosis was psychosis (over 60%); depression was the second highest diagnosis (20%). Mean age in 1996 was 60.8 (range = 45 to 86) and 47% of the sample were women. Only 36% of the group had more than nine-year compulsory education. Compared with other groups in the larger study, this group was more likely to have never married (66%) and 18% had accumulated more than 10 years in an institution over their lifetime (mean = 6 years). Even after correcting for age, functioning as measured by the Global Assessment of Functioning and a 9-item measure assessing hygiene, economy, food preparation, etc. declined over time. As a result, although 49% were living independently (with or without support) at the first wave, only 34% were doing so at the last wave; by 2011 66% were living in a special home or institution. Examination of longitudinal trends in income indicated that disposable income and total income from all sources was constant between 1990 and 1995 but increased significantly over time after reform was enacted. Overall, results suggest mixed success of social support services.

